# Triptolide Affects the Function of Hepatocellular Drug Uptake Transporter Organic Anion Transporting Polypeptide 1B1 Through the Suppression of SGK1

**DOI:** 10.3390/biology14111618

**Published:** 2025-11-18

**Authors:** Zichong Li, Chaomin Pan, Jieru Chen, Xiaoyu Shuai, Mei Hong

**Affiliations:** 1 Guangdong Laboratory for Lingnan Modern Agriculture, College of Life Sciences, South China Agricultural University, Guangzhou 510642, China; 2 Guangdong Provincial Key Laboratory for the Development Biology and Environmental Adaptation of Agricultural Organisms, South China Agricultural University, Guangzhou 510642, China

**Keywords:** OATP1B1, triptolide, transport function, SGK1, NFκB

## Abstract

Organic anion transporting polypeptide 1B1 (OATP1B1) is specifically expressed at the basolateral membrane of human liver cells and transports a wide range of endogenous compounds, toxins, and drugs, making it a crucial factor in determining the pharmacokinetics of many clinically important medications. *Triptergium wilfordii* Hook. f. (TWHF) is a traditional Chinese medicine known for its long history of therapeutic effects. A previous study conducted in our laboratory found that major components of TWHF, including wilforine (WFR), wilforgine (WFG), celastrol (CL), and triptolide (TPL), directly suppressed the function of OATP1B1. In the current study, we investigated the long-term (24 h) effects of these TWHF components on the transporter. It was found that TPL was the most potent compound exhibiting inhibitory effects. Mechanistically, TPL accelerated the degradation of OATP1B1, which is likely mediated by serum and glucocorticoid-induced kinase 1 (SGK1). TPL downregulated the mRNA expression of *SGK1* and reduced the nuclear accumulation of nuclear factor kappa B (NFκB). Further analysis of the upstream sequence of *SGK1* identified three potential binding sites for NFκB. Both luciferase activity assays and chromatin immunoprecipitation (ChIP) analyses confirmed the binding of NFκB to two specific sites located at −1015 bp~−1006 bp and −319 bp~−310 bp.

## Simple Summary

Organic anion transporting polypeptide 1B1 (OATP1B1) is a liver-specific drug transporter that plays a crucial role in the uptake of many clinically significant drugs. In our previous study, the major components of *Tripterygium wilfordii* Hook. f. (TWHF), including wilforine (WFR), wilforgine (WFG), celastrol (CL), and triptolide (TPL), were demonstrated to inhibit the function of OATP1B1. Here, the long-term effect of these compounds on OATP1B1 was investigated. TPL was shown to be the most potent component in such a treatment, suppressing both the protein level and function of OATP1B1 at a concentration as low as 1 nM. It was shown that TPL accelerates the degradation of OATP1B1 by regulating serum and glucocorticoid-induced kinase 1 (SGK1), whose transcript activity was downregulated by the reduced nuclear accumulation of nuclear factor kappa B (NFκB) caused by TPL. Our findings suggest that herbal compounds can lead to herb–drug interactions not only by altering transporter function, but also by affecting protein levels through post-transcriptional regulations.

## 1. Introduction

Organic anion transporting polypeptides (OATPs), represented by the gene symbol *SLCO*, are transmembrane proteins that belong to the solute carrier (SLC) superfamily. They facilitate the sodium-independent transport of various structurally diverse compounds [1]. Among the OATP family members, OATP1B1, which is predominantly expressed at the basolateral membrane of human hepatocytes, plays a crucial role in the clearance of drugs from the body [2]. The substrates of OATP1B1 include endogenous compounds such as bile salts, bilirubin, steroid hormone metabolites, thyroid hormones and their metabolites, as well as inflammatory mediators. It also transports a wide range of clinically significant therapeutic agents, such as statins, sartans, antibiotics, antiviral drugs, and anticancer drugs [3]. This makes OATP1B1 a key factor in determining the pharmacokinetics of these drugs and highlights its importance as a crucial site for drug–drug interactions (DDIs) [4].

Concerns about potential herb–drug interactions (HDIs) are growing due to the increasing use of Chinese herbal medicines (CHMs) as complementary therapies and dietary supplements. HDIs can alter the effects of prescription medications, leading to unexpected clinical outcomes [5]. As membrane transporters play crucial roles in the absorption, distribution, and elimination of important drugs, the modulation of these drug transporters has been recognized as a primary cause of HDIs [5]. For example, compounds such as silymarin, silybin A, silybin B, and silychristin, which are derived from milk thistle (*Silybum marianum*), have been shown to inhibit the uptake of estradiol-17β-glucuronide and rosuvastatin in human liver cells, a process mediated by OATPs [6]. Since milk thistle is a popular herbal supplement increasingly used for self-treating conditions like Type 2 diabetes mellitus and nonalcoholic steatohepatitis (NASH), the silymarin–drug interaction involving OATPs may affect the pharmacokinetics of co-administered drugs [7].

*Triptergium wilfordii* Hook. f. (commonly known as Thunder God Vine, TWHF) is a traditional Chinese medicine with a long history of use that can be traced back to more than two thousand years ago, when it was used for the treatment of edema, fever, chills, sores, carbuncle, and joint pain [8]. The chemical components found in TWHF include alkaloids, diterpenoids, and triterpenoids [9]. In our previous study [10], it was found that the co-incubation of sesquiterpene pyridine alkaloids wilforine (WFR) and wilforgine (WFG) exhibited a potent inhibitory effect on the uptake of 2′, 7′-dichlorofluorescein (DCF) and pitavastatin by OATP1B1. The co-incubation resulted in reduced values for both K_m_ and V_max_ for DCF uptake. Conversely, a short-term pre-incubation (less than 3 h) of WFR and WFG led to increased K_m_ but no change in V_max_ for the uptake of DCF. Additionally, the triterpenoid celastrol (CL) also demonstrated an inhibitory effect on OATP1B1 transport function, resulting in decreased K_m_ and V_max_ values in both co-incubation and short-term pre-incubation treatments. However, the diterpenoid triptolide (TPL) showed a significantly weaker effect on OATP1B1 compared to the other compounds in these treatments. Triptolide (TPL) is recognized as the most potent bioactive component of TWHF. It exhibits immunosuppressive, anti-inflammatory, and anti-tumor effects. However, despite the growing therapeutic applications of TPL in recent years, its toxicity poses a significant challenge to its use. The liver is one of the primary organs impacted by TPL-induced toxicities [11]. In the present study, TPL was identified to be the most potent TWHF component that inhibited the uptake function of OATP1B1 after long-term exposure. Further analysis revealed that TPL may accelerate the degradation of OATP1B1 through its influence on serum and glucocorticoid-induced kinase 1 (SGK1). It was found that TPL suppressed *SGK1* expression by reducing the nuclear accumulation of nuclear factor kappa B (NFκB).

## 2. Materials and Methods

### 2.1. Materials

Reagents and enzymes for molecular biology and cell culture were obtained from Thermo Fisher Scientific (Waltham, MA, USA). Triptolide, celastrol, wilforgine, and wilforine were purchased from Chengdu Pufei De Biotech Co., Ltd. (Chengdu, China). Antibodies for the detection of HA (#AF0039) and NFκB (#AF0246) were purchased from Beyotime Biotech Inc. (Shanghai, China). Anti-SGK1 (#A1025) was obtained from Abclonal (Woburn, MA, USA). All other chemicals were sourced from Sigma (St. Louis, MO, USA) unless otherwise specified.

### 2.2. Cell Culture of HEK293 Cells Stably Overexpressing Human SLCO1B1

Human embryonic kidney 293 (HEK293) cells, which stably express human *SLCO1B1* with 3 × HA tags attached to the C-terminus (designated as HEK293-OATP1B1), were generated and characterized in our previous studies [10]. The cells were maintained at 37 °C with 5% CO_2_ in Dulbecco’s modified Eagle’s medium, supplemented with 10% fetal bovine serum and 0.5 mg/mL of G418.

### 2.3. Uptake Assay

Cells were seeded into 48-well plates and incubated for 24 h. Afterward, various TWHF compounds were added. Transport measurements were performed 24 h later, following thorough washing of the incubated cells with warmed (37 °C) uptake solution without the substrate. The uptake solution that contained 2′,7′-dichlorofluorescein (DCF) was then added. To stop the uptake process, ice-cold phosphate-buffered saline (PBS) was added, and the cells were washed twice more with cold PBS. Subsequently, the cells were solubilized with NaOH, and detection was carried out using a SpectraMax i3x Multi-Mode Microplate Reader (Molecular Devices, San Jose, CA, USA). The uptake counts were standardized based on the protein concentration in each well.

### 2.4. RNA Isolation and RT-PCR

Total RNA was extracted from cells using TRIzol^®^ reagent (Thermo Fisher Scientific). An equal amount of RNA was used for reverse transcription to synthesize complementary DNA (cDNA). The resulting cDNA was then analyzed using real-time quantitative polymerase chain reaction (qPCR). The primers for *SGK1* (Accession # NM_005627) were forward 5′-GGTGGCAATTCTCATCGCTTT-3′ and reverse 5′-ACTTGGTGGAGGAGAAGGG T-3′. Actin served as the internal control, and primers for the detection of actin level were forward 5′-TGGGCATGGGTCAGAAGGAT-3′ and reverse 5′-GTGTGGACTTGGGA GAGGAC-3′. At the end of each qPCR reaction, dissociation curves of the products were analyzed to confirm the specificity of each gene amplification. The relative quantification of the target mRNAs was calculated by normalizing against the actin endogenous reference.

### 2.5. Construction of Plasmid DNA with Various Fragments of the Upstream Regulatory Sequence of SGK1

Different fragments of the upstream regulatory sequence of SGK1 were incorporated into the pGL3 basic luciferase reporter vector using the Omega-PCR technique [12] with the primers listed in Table 1. Site-directed mutagenesis was performed using the QuikChange Lightning Site-Directed Mutagenesis Kit from Agilent (Santa Clara, CA, USA). The sequences of all mutant constructs were confirmed through full-length sequencing.

### 2.6. Analysis of Luciferase Activity

Luciferase activity was measured using the Dual-Luciferase Reporter Assay System (Promega, Madison, WI, USA) in accordance with the manufacturer’s instructions. Briefly, cells grown in 48-well plates were lysed with passive lysis buffer. After lysis, Luciferase Assay Reagent II was added, and the samples were analyzed with a GloMax™-Multi Jr Single Tube Multimode Reader (Promega). Following this, Stop & Glo™ Reagent in the Dual-Luciferase Reporter Assay System was added to quench the luminescence of firefly luciferase while simultaneously activating the Renilla luciferase. The activity of firefly luciferase was normalized by the activity of the Renilla luciferase, and the values of the DMSO-treated control were designated as 100% in each set of experiments.

### 2.7. Chromatin Immunoprecipitation (ChIP)

Chromatin immunoprecipitation was performed using the ChIP assay kit from Beyotime Biotechnology Inc. (Shanghai, China), following the methodology outlined by Xiang et al. [13]. In brief, cells were cross-linked with 1% formaldehyde at room temperature for 10 min. The cross-linking reaction was quenched using glycine, after which the cells were scraped, washed, and lysed with SDS lysis buffer. The solution was then subjected to sonication with a Branson S450-D digital sonifier (Branson Ultrasonic, Danbury, CT, USA). An antibody diluted to 1:100 was added to the solution and incubated overnight at 4 °C to label the corresponding proteins. The proteins were subsequently captured using protein G agarose/salmon sperm DNA. After releasing the DNA–protein complexes from the beads, the DNA was purified using the High Pure PCR Product Purification Kit (Roche Applied Science, Penzberg, Germany) and analyzed using qPCR. The primers used for the analysis were as follows: For site 1 (−1015 bp to −1006 bp), the forward primer was 5’-CCACATTCCTGACCTCTCC-3′ and the reverse primer was 5′-CTTGTGCACGGTCGGGTC-3′. For site 2 (−319 bp to −310 bp), the forward primer was 5′-GACAGTGAGCGAAGCCACC-3′ and the reverse primer was 5′-GTTATCAGTCTCCATCGGCT-3’. The DNA amplified from the NFκB antibody pull-down was normalized to that amplified from the total DNA in the same reaction system.

### 2.8. Statistical Analysis

The Student’s *t*-test was used to compare two sets of data. One-way analysis of variance (ANOVA) was utilized for comparisons involving more than two groups. Differences between means were considered significant if *p* < 0.05.

## 3. Results

### 3.1. Long-Term Incubation of TWHF Components Affected Uptake Function of OATP1B1

**Figure 1:**
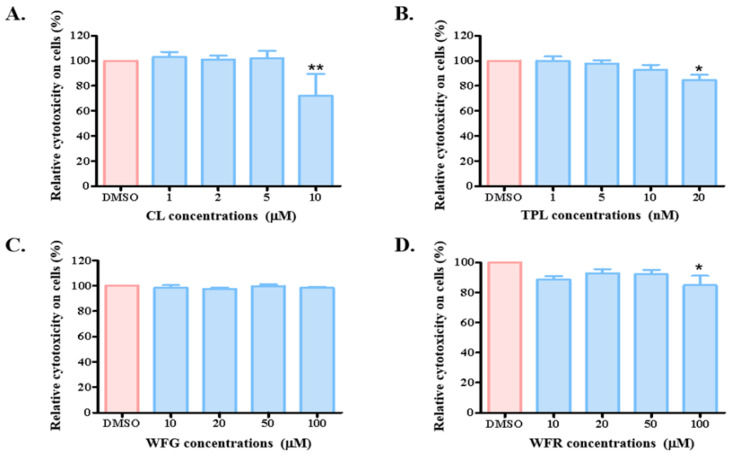
Cytotoxicity of different TWHF components on OATP1B1-expressing cells. HEK293-OATP1B1 cells were treated with indicated concentrations of CL (**A**), TPL (**B**), WFG (**C**), and WFR (**D**) for 24 h. Cell viability was then quantified using the MTT assay, in which formazan, the metabolized form of MTT, was measured at 450 nm. The readings of treated samples (represented as blue columns) were normalized to the DMSO-treated control (designated as 100% viability in each set of experiments and represented as red columns). The results represent data from three independent experiments, with triplet measurements for each sample. The results are shown as means ± SEM (*n* = 3). Asterisks indicate significantly different from the control (* *p* < 0.05, ** *p* < 0.01).

As our purpose was to evaluate the effect of TWHF components on the activity of OATP1B1 under conditions that do not cause other deleterious effects on the cells, we first analyzed the cytotoxicity of CL, TPL, WFG, and WFR on HEK293-OATP1B1 cells. As shown in Figure 1, after incubating HEK293-OATP1B1 cells with different concentrations of TWHF components for 24 h, CL exhibited no toxic effect at concentrations below 5 μM. The cells remained well-tolerant to the two alkaloids, showing reduced cell survival only when exposed to 100 μM of WFR. On the other hand, TPL demonstrated cytotoxicity at a concentration as low as 20 nM.

**Figure 2:**
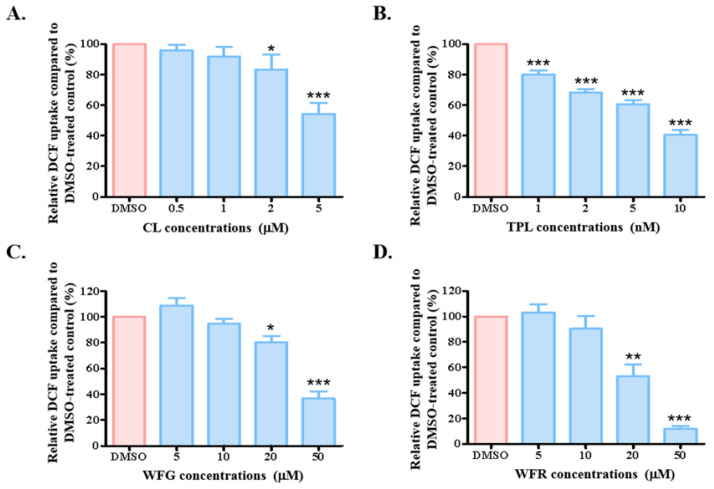
Effect on OATP1B1 function after long-term treatment with TWHF components. Cells overexpressing OATP1B1 or empty vector control were treated with CL (**A**), TPL (**B**), WFG (**C**), and WFR (**D**) for 24 h. The cells were then washed thoroughly with uptake solution before the DCF uptake was measured at 37 °C for 5 min. Transport activity of TWHF component-treated cells (represented as blue columns) was calculated as a percentage of uptake by the DMSO-treated control cells (designated as 100% function in each set of experiments and represented as red columns). The results represent data from three independent experiments, with duplicate measurements for each sample. The results shown are the means ± SEM. (*n* = 3). Asterisks indicate significantly different from control (* *p* < 0.05, ** *p* < 0.01, *** *p* < 0.001).

After identifying the concentrations that did not cause toxic effects on HEK293-OATP1B1 cells, we proceeded to measure the uptake of DCF by the cells. Our results showed that CL, along with WFG and WFR, significantly inhibited OATP1B1 activity at micromolar levels. Notably, TPL began to exhibit a suppressive effect at a concentration as low as 1 nM (Figure 2). Given that TPL demonstrated the most potent inhibitory effect on OATP1B1 function, we further explored the underlying mechanism behind this effect.

### 3.2. TPL Accelerated the Degradation of OATP1B1

**Figure 3:**
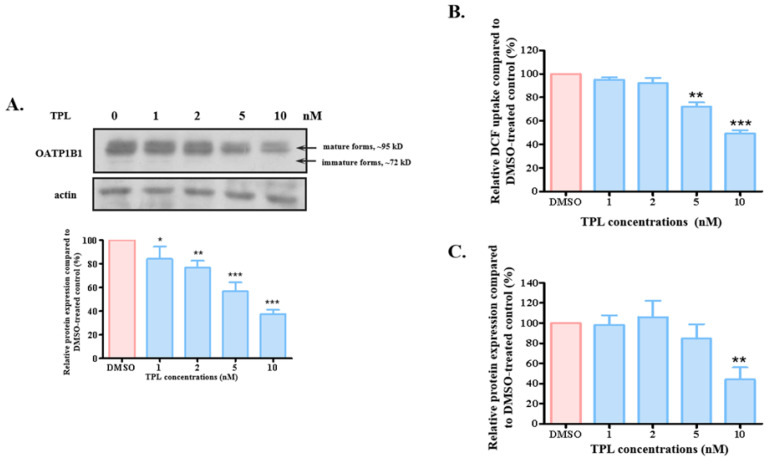
Triptolide reduced the protein level of OATP1B1. (**A**) Effect of TPL on protein expression of OATP1B1 in HEK293-OATP1B1 cells. (**B**) Uptake of DCF by OATP1B1 in HepG2 cells after being treated with different concentrations of TPL for 24 h. (**C**) Effect of TPL on protein level of OATP1B1 expressed in HepG2 cells. OATP1B1-expressing cells were treated with different concentrations of TPL for 24 h (**A**,**C**). Proteins were extracted, denatured, and separated by SDS-PAGE, followed by Western blotting with anti-HA antibody (1:1000 dilution). Same blot was probed with actin antibody as loading control. Intensity of protein bands in treated samples (represented as blue columns) was quantified with Image J (version 1.54p) and compared relative to the protein level in the DMSO-treated control (designated as 100% in each set of experiments and represented as red columns). Transport activity of TPL-treated (represented as blue columns) cells was calculated as a percentage of uptake by the DMSO-treated control cells (designated as 100% function in each set of experiments and represented as red columns) (**B**). The results represent data from three independent experiments, with duplicate measurements for each sample. The results shown are the means ± SEM. (*n* = 3). Asterisks indicate significantly different from the control (* *p* < 0.05, ** *p* < 0.01, *** *p* < 0.001).

The 24 h treatment with TPL may affect the function of OATP1B1 by influencing its expression level. When the protein level of OATP1B1 was measured, it was observed that the transporter level decreased with increasing concentrations of TPL, showing a marginal but significant reduction at as low a concentration as 1 nM (Figure 3A and Figure S1 for original blots). Treatment with TPL at 10 nM resulted in approximately 60% reduction in the protein level of OATP1B1, which correlated well with the decrease in its uptake. To rule out the possibility that this effect is specific to the cell type, we measured DCF uptake and the protein level in HepG2 cells overexpressing OATP1B1 after treatment with different concentrations of TPL. It was found that TPL also suppressed the transport function and protein expression of OATP1B1 in the HepG2 cells, but the responses were less pronounced compared to what was observed in the HEK293 cells (Figure 3B,C). Consequently, we conducted further experiments using HEK293-OATP1B1 cells.

**Figure 4:**
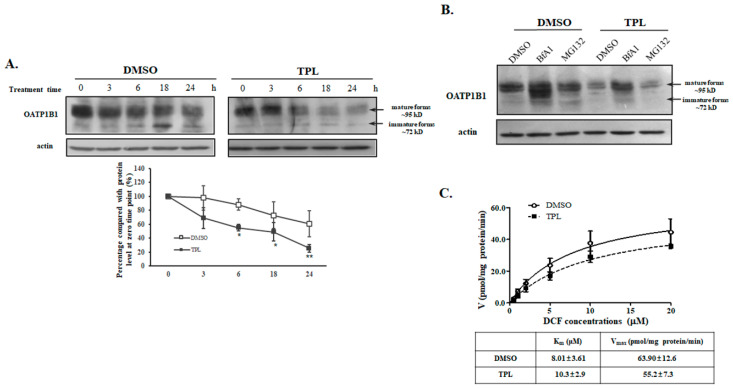
Triptolide treatment accelerated the degradation of OATP1B1. (**A**). Cyclohexamide-chase analysis of TPL-treated cells. (**B**). Effect of proteasomal and lysosomal inhibitors on TPL-treated cells. Cells treated with 10 nM TPL together with 100 μg/mL of cycloheximide were collected at indicated time points (**A**), or 10 nM TPL together with 20 μM MG132 or 100 nM BfA1 were applied to cells for 24 h (**B**). A representative blot is shown. Intensity of protein bands was quantified with ImageJ and compared relative to the original protein level at the beginning of the treatment (designated as 100% in each set of experiments). The results shown are the means ± SEM (*n* = 3). Asterisks indicate significant difference compared to the DMSO-treated control (* *p* < 0.05, ** *p* < 0.01). (**C**) DCF transport kinetics by TPL-treated cells. HEK293-OATP1B1 cells were pre-incubated with TPL (10 nM) for 24 h. After the cells were thoroughly washed, DCF uptake was measured at concentrations ranging from 0.5 to 20 µM at 37 °C for 2 min and normalized with the corresponding relative protein level of OATP1B1. The results represent data from three independent experiments, with duplicate measurements for each sample. The results shown are the mean ± SEM (*n* = 3). K_m_ and V_max_ were calculated using the nonlinear regression of the Michaelis–Menten equation incorporated in GraphPad Prism 8.

Further analysis using the protein synthesis inhibitor cycloheximide revealed that TPL treatment significantly increased the degradation rate of OATP1B1 (Figure 4A and Figure S2 for original blots). Since membrane proteins are typically degraded through proteosomal and/or lysosomal pathways, we treated the cells with the proteasomal inhibitor MG132 and bafilomycin A1 (BfA1), a potent lysosomal inhibitor. We found that only BfA1 could partially recover the protein level of OATP1B1 (Figure 4B and Figure S2 for original blots). Additionally, we measured the uptake of different concentrations of DCF by OATP1B1 at the presence of TPL, and normalized this to the level of OATP1B1. The results indicate that neither the K_m_ nor the V_max_ value showed any significant changes after TPL treatment (Figure 4C). These findings suggest that the primary effect of TPL on OATP1B1 is related to its impact on the degradation of the transporter protein.

### 3.3. SGK1 Is Involved in the Effect of TPL on OATP1B1

**Figure 5:**
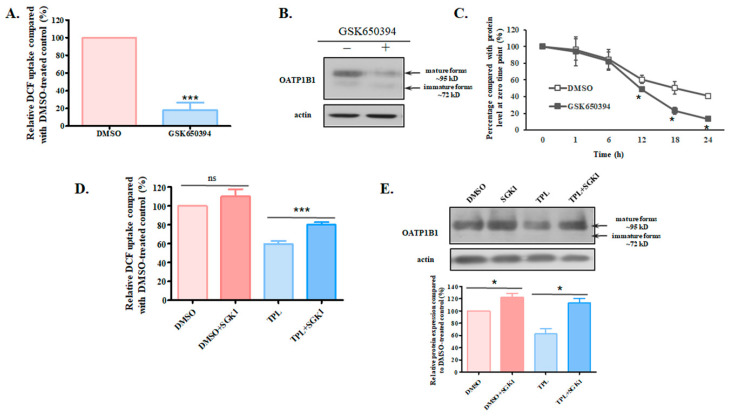
SGK1 is involved in the degradation of OATP1B1 by TPL. (**A**) DCF uptake by HEK293-OATP1B1 cells treated with SGK1 inhibitor GSK650394. (**B**) Protein expression of OATP1B1-expressing cells treated with GSK650394. Cells were treated with 10 μM GSK650394 for 24 h, and DCF uptake or protein level was measured as described above. (**C**) Degradation kinetics of OATP1B1 after GSK650394 treatment. OATP1B1-expressing cells were treated with GSK650394 together with cycloheximide and collected at the indicated time points. Proteins were extracted and analyzed as described in Figure 4. The intensity of protein bands was quantified with ImageJ and compared relative to the original protein level at the beginning of the treatment (designated as 100% in each set of experiments). (**D**) DCF uptake by HEK293-OATP1B1 cells overexpressing SGK1. (**E**) Protein level of OATP1B1 in cells overexpressing SGK1. Cells were transfected with the SGK1 expression vector 24 h after plating. Twenty-four hours after transfection, TPL was added to the culture medium, and cells were treated for an additional 24 h before DCF uptake (**D**) or OATP1B1 protein level (**E**) was measured. The results shown are the means ± SEM (*n* = 3). Asterisks indicate significant difference compared to the control (* *p* < 0.05, *** *p* < 0.001). ns stands for not significant.

TPL has been found to affect various signaling pathways, including the phosphoinositide 3-kinase (PI3K)/Akt pathway [14,15]. However, we found that the inhibition of Akt exerted no effect on the function of OATP1B1. On the other hand, the inhibition of SGK1, another downstream effector of PI3K [16], led to significantly reduced function and protein expression of the transporter (Figure 5A,B and Figure S3 for original blots). Furthermore, treatment with the SGK1 inhibitor GSK650394 accelerated the degradation of the transporter, a phenomenon similar to that observed with TPL treatment (Figure 5C). To see whether SGK1 contributed to the TPL-induced effect on OATP1B1, we overexpressed SGK1 in HEK293-OATP1B1 cells. It was found that the overexpression of SGK1 significantly mitigated the impact of TPL on OATP1B1, leading to increased uptake function (Figure 5D) and elevated protein level (Figure 5E and Figure S3 for original blots) of the transporter.

### 3.4. TPL Downregulated SGK1 Through NFκB

**Figure 6:**
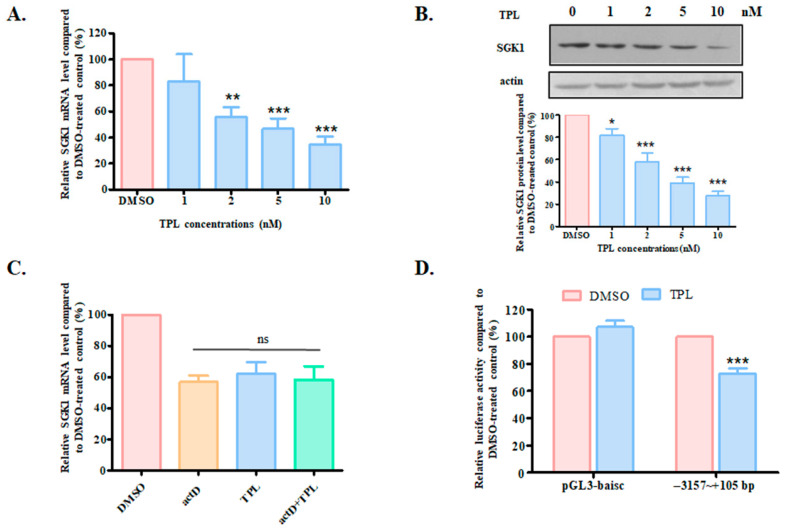
Triptolide suppressed the transcription of SGK1. mRNA (**A**) and protein (**B**) levels of SGK1 after TPL treatment. Cells treated with different concentrations of TPL for 24 h and then collected for RNA or protein extraction. Total RNA was extracted and subjected to reverse transcription reaction and quantitative PCR. (**C**) ActD treatment of HEK293-OATP1B1 cells. Cells were treated with TPL with or without 10 μM ActD. Total RNA was extracted and analyzed. (**D**) TPL effect on the luciferase activity of the 3 kb upstream regulatory sequence of *SGK1*. Luciferase activity was measured and calculated relative to that of the Renilla luciferase activity. The results of the TPL-treated samples (represented as blue columns) were expressed as a percentage to that of the DMSO-treated control, which was designated as 100% in each set of experiments and represented as red columns. The results represent data from three independent experiments and were expressed as the mean ± SEM (*n* = 3). Asterisks indicate significant difference compared to the control (* *p* < 0.05, ** *p* < 0.01, *** *p* < 0.001). ns stands for not significant.

To see what kind of effect TPL imposed on SGK1, we measured both the mRNA and protein levels of SGK1 after TPL treatment. As shown in Figure 6A,B (Figure S4 for original blots), SGK1 was dose-dependently downregulated by TPL, with 1 nM of TPL already showing a downward trend effect on the expression of the kinase. Concurrent treatment of TPL with actinomycin D, an inhibitor of RNA synthesis, exhibited a similar effect on the SGK1 mRNA level as the treatment with TPL alone (Figure 6C), suggesting that the suppressive effect of TPL on SGK1 expression occurs at the transcription level. To further verify the influence of TPL on SGK1 transcription, a luciferase expression vector that contains the 3 kb upstream sequence of the *SGK1* gene was generated, and the luciferase activity was measured. As demonstrated in Figure 6D, TPL treatment significantly suppressed the luciferase activity.

**Figure 7:**
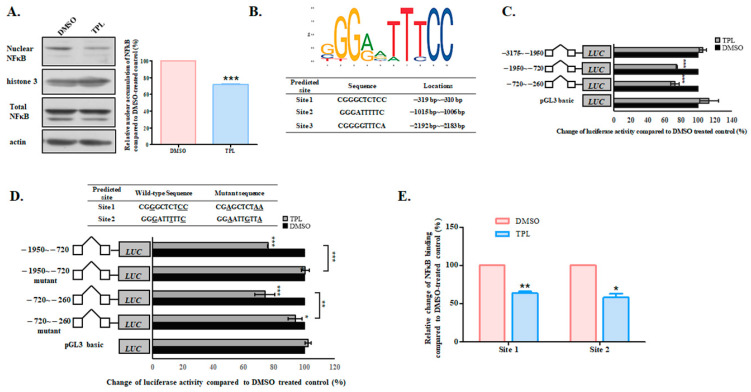
NFκB is involved in the TPL suppression of SGK1. (**A**) Triptolide suppressed NFκB nuclear accumulation. Nuclear and total proteins were extracted from TPL-treated cells and subjec ted to Western blotting analysis. Nuclear NFκB level was normalized to the total NFκB level and expressed as a percentage of that of the cells treated with DMSO (designated as 100% in each set of experiments). Three independent experiments were performed. The data were expressed as the mean ± SEM. (*n* = 3). Asterisks indicate values significantly different (*** *p* < 0.001). (**B**) Conserved bases of the NFκB binding site (**upper panel**) and predicted NFκB binding sites (**lower panel**) in the upstream sequence of *SGK1*. (**C**) Luciferase activity of constructs containing different fragments of the upstream regulatory region of *SGK1*. (**D**) Luciferase activity for mutants of the predicted NFκB binding sites after TPL treatment. The mutated bases are underlined. Luciferase activity was measured and calculated relative to that of the Renilla luciferase activity. The results were expressed as a percentage compared to that of the cells treated with DMSO, which was designated as 100% in each set of experiments. Three independent experiments were performed, each with duplicate measurements. The data were expressed as the mean ± SEM (*n* = 3). Asterisks indicate values significantly different (* *p* < 0.05, ** *p* < 0.01, *** *p* < 0.001) from the control. (**E**) ChIP analysis for the interaction of NFκB with the putative binding sites of *SGK*1. The cross-linked DNA/protein complexes were precipitated with the NFκB antibody and subjected to qPCR for the detection of site 1 and site 2. The results were expressed as a percentage compared to that of the cells treated with DMSO, which was designated as 100% in each set of experiments. Three independent experiments were performed. The data were expressed as the mean ± SEM (*n* = 3). Asterisks indicate values significantly different (* *p* < 0.05, ** *p* < 0.01) from that of the DMSO-treated control. The DMSO-treated results are represented as red columns, and the TPL-treated samples are represented as blue columns.

Since TPL is involved in cellular inflammatory responses, the diterpenoid has been reported to alter the expression of various genes through the inflammation-related transcription factor NFκB [17,18,19]. Consequently, we aimed to investigate whether TPL suppressed SGK1 via NFκB. Our findings revealed that while the total amount of NFκB remained unchanged, the nuclear accumulation of this transcription factor significantly decreased following TPL treatment (Figure 7A and Figure S5 for original blots). An analysis of the *SGK1* upstream 3 kb sequence using JASPAR (https://jaspar.genereg.net) identified three potential NFκB binding sites (Figure 7B). We then divided the upstream 3 kb sequence into three smaller fragments, each containing one predicted NFκB binding site. As shown in Figure 7C, only the fragments containing sites 1 and 2 exhibited a significant reduction in activity after TPL treatment. In contrast, the fragment with site 3 showed no changes following the terpenoid treatment. We also mutated the highly conserved bases in sites 1 and 2 and observed that the response to TPL was significantly impaired in the mutants (Figure 7D). This suggests that NFκB directly binds to these two sites to exert its effect on *SGK1*. To confirm that TPL treatment indeed affected NFκB binding to sites 1 and 2, we performed a ChIP assay using an NFκB antibody. The analysis of quantitative PCR coupled with ChIP indicated that TPL significantly suppressed the formation of endogenous DNA/NFκB complexes (Figure 7E).

### 3.5. Overexpression of NFκB Attenuated the Effect of TPL on OATP1B1 Protein Expression and Function

**Figure 8:**
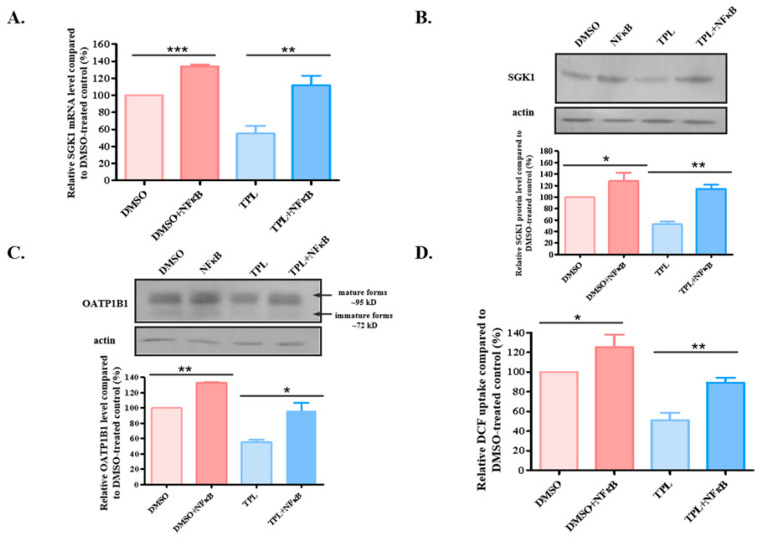
Overexpression of NFκB attenuated TPL suppression of OATP1B1. mRNA (**A**) and protein (**B**) levels of SGK1 in cells overexpressing NFκB. (**C**) Protein level of OATP1B1 in cells overexpressing NFκB. (**D**) DCF uptake by HEK293-OATP1B1 cells overexpressing NFκB. Cells were transfected with the NFκB expression vector 24 h after plating. Twenty-four hours after the transfection, TPL was added to the culture media and treated for another 24 h before the level of mRNA (**A**), protein (**B**,**C**), or DCF uptake (**D**) was measured. The results were expressed as a percentage compared to that of the cells treated with DMSO, which was designated as 100% in each set of experiments. The results shown are the means ± SEM (*n* = 3). Asterisks indicate significant difference compared to the control (* *p* < 0.05, ** *p* < 0.01, *** *p* < 0.001).

The above results indicate that TPL may downregulate the expression of *SGK1* though its suppression on NFκB nuclear accumulation, and the reduced level of SGK1 led to accelerated degradation of OATP1B1. To validate the functional cassette, we overexpressed NFκB in HEK293-OATP1B1 cells. As shown in Figure 8, the overexpression of NFκB significantly increased the mRNA and protein levels of SGK1 (Figure 8A,B and Figure S6 for original blots), which also significantly attenuated the effect of TPL on OATP1B1 protein level and uptake function (Figure 8C,D and Figure S6 for original blots).

## 4. Discussion

The increasing use of herbs as alternative and/or supplemental medicine has attracted much attention in recent years. Although herbal-related consumption may improve the therapeutic outcome of certain traditional medicines, it was also demonstrated to affect the pharmacokinetics and/or pharmacodynamics of many clinically important drugs. OATP1B1 is a well-recognized determinant for the absorption, distribution, and excretion of multiple drugs and an important DDI site [20]. Quite a few studies have demonstrated that herbal components directly inhibit OATP1B1 function [21]. However, whether the function and/or protein level of this transporter is affected after low doses and long-term exposure to herbal compounds is largely unknown. In the present study, we evaluated the influence of major terpenoids and alkaloids of TWHF on OATP1B1 function after exposing the OATP1B1-expressing cells to the chemicals for an extensive period of time. Concentrations and time periods utilized in the current study exhibited no cytotoxicity of the compounds, assuring that the effect is more relevant to normal but not pathological conditions, which are more prone to be ignored by patients concurrently taking herbal medicines with traditional therapeutic agents.

Our previous study concerning the effect of TWHF components on OATP1B1 demonstrated that TWHF alkaloids WFG and WFR not only directly inhibit the uptake function of OATP1B1, but also exhibited a suppressive effect when OATP1B1-expressing cells were exposed to the alkaloids for a short-term period (less than 3 h) before the uptake function was measured. Such an effect was found to be related to the induced phosphorylation level of OATP1B1, and the K_m_ for DCF uptake by OATP1B1 was significantly increased, suggesting that, in addition to directly inhibiting OATP1B1 function, WFG and WFR can also alter the conformation of OATP1B1 through post-translational modification [10]. However, in our previous study, TPL exhibited a much less potent effect than other TWHF components tested; the IC_50_ was 100-fold higher than that for the alkaloids in the co-incubation treatment, while no inhibitory effect was observed when a TPL concentration as high as 100 μM was used to pre-incubate the OATP1B1-expressing cells. These results suggest that TPL is not a direct inhibitor of OATP1B1. On the other hand, our present study found that long-term TPL treatment led to accelerated degradation of OATP1B1 through its effect on SGK1, a downstream target of the PI3K pathway, revealing a novel mechanism that alters the transport activity of OATP1B1 by a TWHF component. The reduced activity of SGK1 led to the accelerated degradation of OATP1B1, and the similarity in the phenomenon led us to postulate that SGK1 is involved in the effect of TPL on OATP1B1. Indeed, the overexpression of SGK1 partially recovered the protein level and activity of OATP1B1 after TPL treatment. SGK1 has a short half-life for mRNA, and the polyubiquitinated protein exhibits a high turnover rate [16]. Additionally, reports also demonstrated that the overexpression of exogenous SGK1 often resulted in a high degree of degradation [22,23]. Therefore, the transcription regulation of SGK1 is of great importance for the level and activity of the kinase. Hence, inhibition of the SGK1 transcript by TPL may greatly alter the availability of the kinase that regulates the protein level of OATP1B1. SGK1 was demonstrated to phosphorylate the ubiquitin ligase Nedd4-2, which in turn reduces its association with OAT3 and the ubiquitination of the transporter, leading to an increased function of the transporter [24]. Whether a similar mechanism occurs in the relationship between SGK1 and OATP1B1 is currently being investigated in our laboratory.

Triptolide has a well-known bioactive effect on inflammatory disorders. Many reports have suggested that it exhibits an inhibitory effect on the NFκB-related pathways. Lower concentrations of TPL are more likely to exert a protective effect by suppressing inflammatory factors such as NFkB, while higher concentrations of the terpenoid often lead to oxidative stress, at which the activity and/or level of Nrf2 is affected [25]. In the present study, it was found that TPL interfered with the nuclear accumulation of NFκB, which in turn downregulated the expression of *SGK1*. TPL was also found to suppress the function and expression level of transporters such as the glucose transporters in cardiomyocytes [26] and efflux transporters such as the P-glycoprotein [27,28] through the inhibition of NFκB. The inhibitory effect of TPL on multidrug resistance-related transporters makes it a potent candidate to circumvent the resistance of cancer cells to chemotherapeutic drugs. However, according to our results, TPL may also affect OATP1B1 function, and the bioavailability of OATP1B1 substrates such as statins is likely to be affected when a residual amount of TPL exists after administration, which needs to be taken into consideration. Triptolide exhibited a relatively potent effect in the long-term treatment, started to show an inhibitory effect at 1 nM, and around a 60% reduction was observed at the concentration of 10 nM. These concentrations are likely to occur in the human liver, at which OATP1B1 is located. It was reported that TPL has a quick absorption and low clearance profile, and the concentration of TPL can reach a C_max_ of around 160 ng/mL in human plasma, with liver showing the highest concentration among the tested organs such as heart, brain, liver, kidney, spleen, intestine, skeletal-muscle, and stomach [29]. Hence, the accumulated concentration of TPL in the liver may reach the concentrations investigated in the present study, and a likely DDI may occur.

However, it should be noted that our current study was conducted using overexpressing cellular systems. While these systems can clearly identify the specific transporter involved in the effects of the herbal compound, they may not accurately represent the in vivo response. Additionally, OATP1B1 lacks a one-to-one counterpart in rodent models, which complicates the evaluation of the actual effects of TPL in vivo. To better assess the herb–drug interaction risk posed by TPL through the mechanisms described, further exploration using humanized mouse or rat models will be necessary. Furthermore, although our study applied a 24 h incubation period for TPL, this duration is still relatively short concerning chronic exposure. Therefore, longer treatment times, particularly in in vivo systems, should be investigated for a more comprehensive understanding of the effects caused by this TWHF component.

## 5. Conclusions

In summary, our present study demonstrated that TPL, the major terpenoid in TWHF, downregulates the transcription of SGK1 through reducing the binding of NFκB with *SGK1* upstream regulatory sites, and the reduced level of SGK1 leads to the accelerated degradation of OATP1B1, which in turn affects the function of the transporter. This novel mechanism suggests that long-term, low-dose exposure to herbal compounds may impact the protein level of transporters rather than their function. Therefore, these types of herb–drug interactions may need to be taken into consideration when using herbal medicines for an extended period.

## Figures and Tables

**Table 1:** Primers for the luciferase reporter constructs with different fragments of the upstream regulatory sequence of *SGK1*.

Fragments	Forward Primer	Reverse Primer
−3157 bp~−1950 bp	CAAGGAGTTGAGACTAGAGCAGC CGATGGAGACTGATAAC	GTTATCAGTCTCCATCGGCTGCT CTAGTCTCAACTCCTTG
−1950 bp~−720 bp	CGAGCTCTTACGCGTGCTAGCCAA GGAGTTGAGACTAGAGC	GCTCTAGTCTCAACTCCTTGGCT AGCACGCGTAAGAGCTCG
−720 bp~−260 bp	CGAGCTCTTACGCGTGCTAGCAG CGAGTCCTTCCTGCTGA	TCAGCAGGAAGGACTCGCTGCT AGCACGCGTAAGAGCTCG

## Data Availability

The original contributions presented in this study are included in the article/Supplementary Materials. Further inquiries can be directed to the corresponding author.

## Supplementary Materials

The following supporting information can be downloaded at: https://www.mdpi.com/article/10.3390/biology14111618/s1, Figure S1: Original blots for Figure 3A; Figure S2: Original blots for Figure 4A,B; Figure S3: Original blots for Figure 5B,E; Figure S4: Original blots for Figure 6B; Figure S5: Original blots for Figure 7A; Figure S6: Original blots for Figure 8B,C.
