# Supplementary material for: Triptolide Affects the Function of Hepatocellular Drug Uptake Transporter Organic Anion Transporting Polypeptide 1B1 Through the Suppression of SGK1
Source: Biology (Basel). 2025 Nov 18;14(11):1618. doi: 10.3390/biology14111618 (PMC12650508; doi:10.3390/biology14111618)

Figure S1 Original blots for Figure 3A

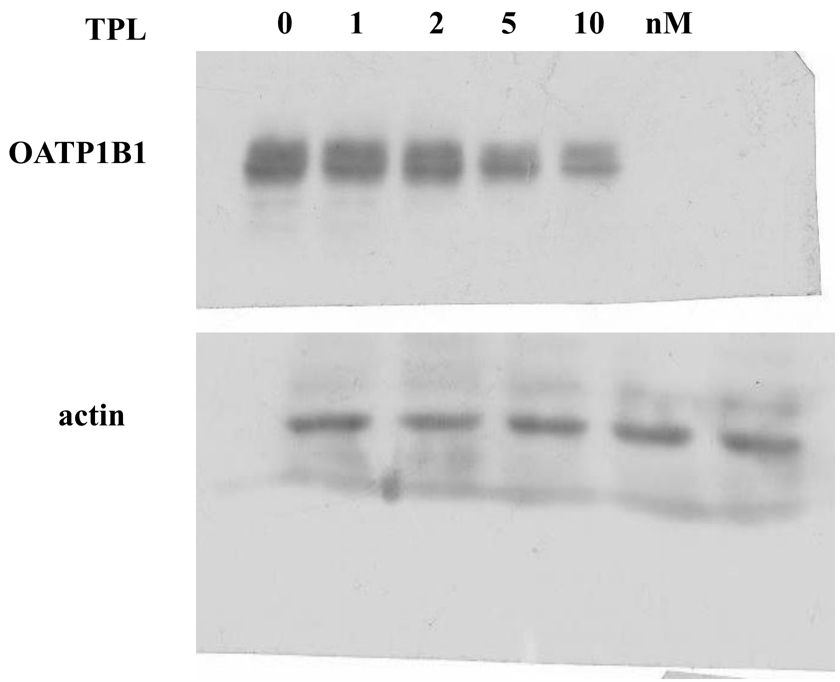

Figure S2 Original blots for Figure 4A and 4B

Figure 4A

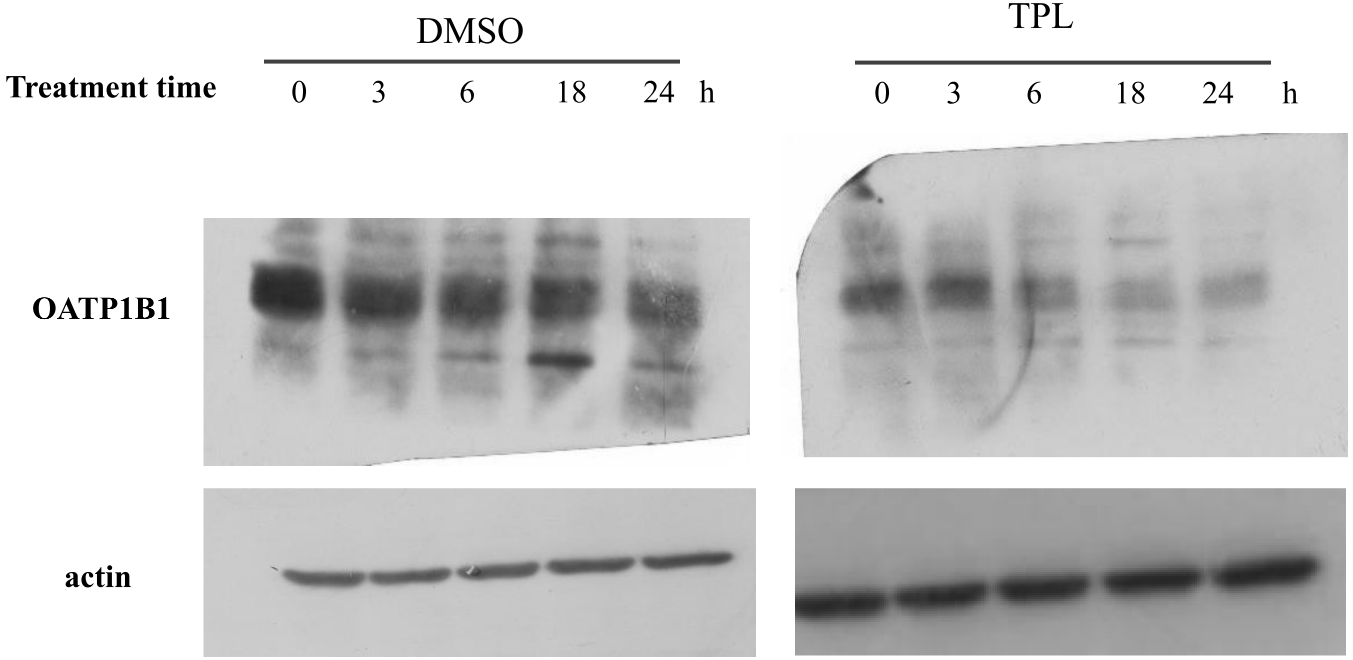

Figure 4B

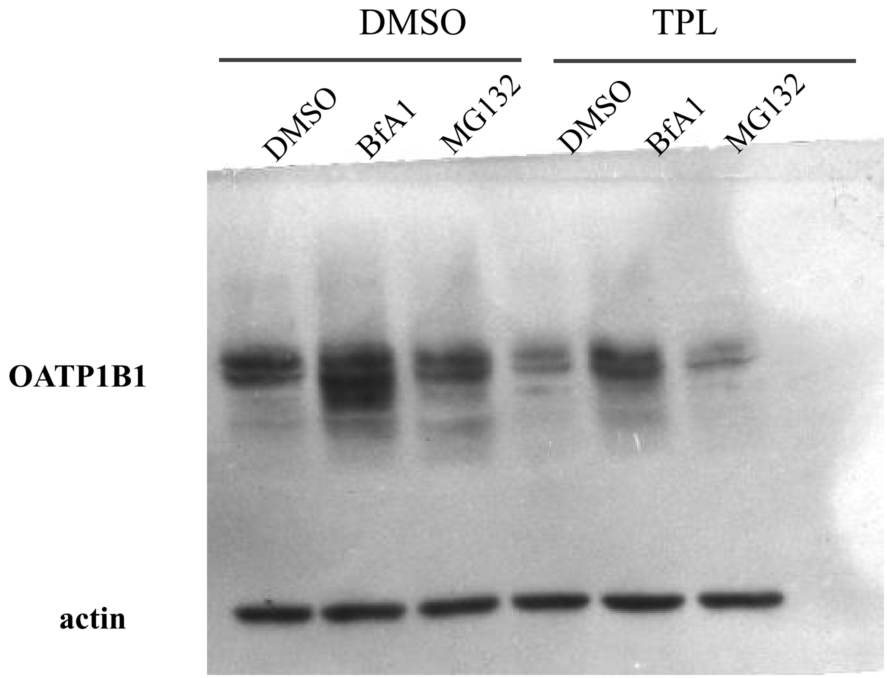

Figure S3 Original blots for Figure 5B and 5E

Figure 5B

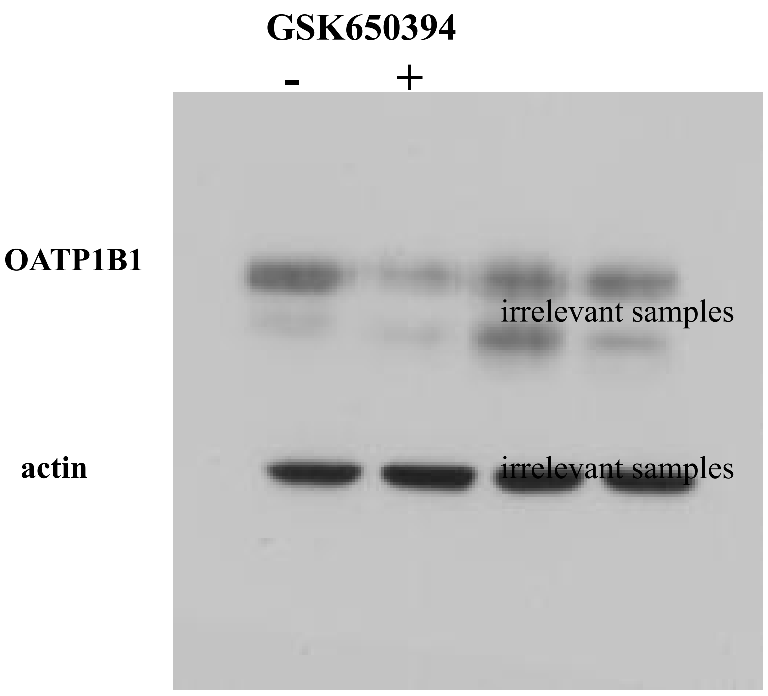

Figure 5E

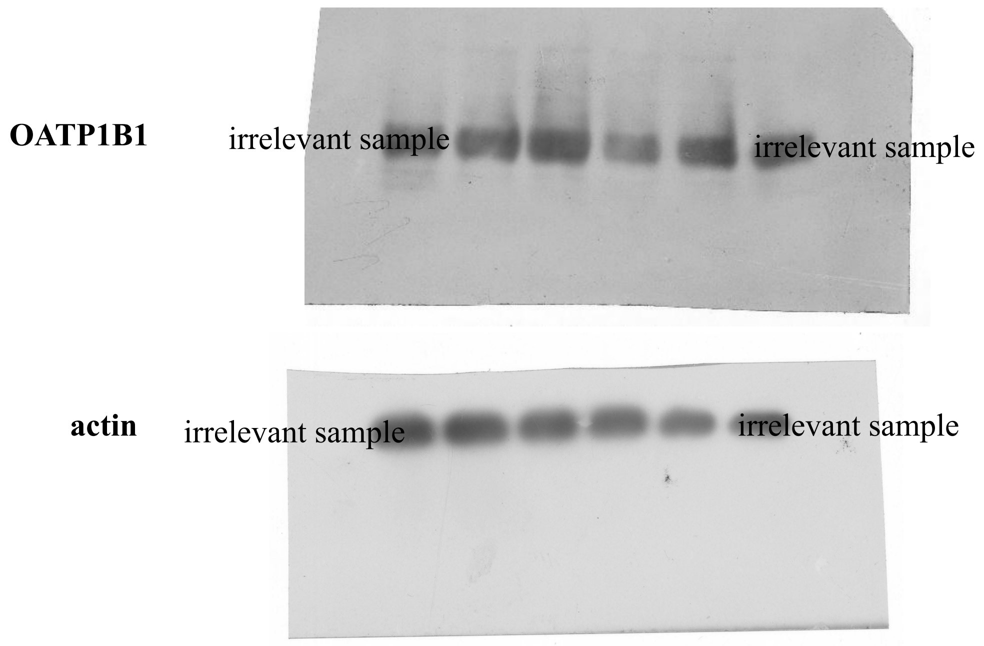

Figure S4 Original blots for Figure 6B

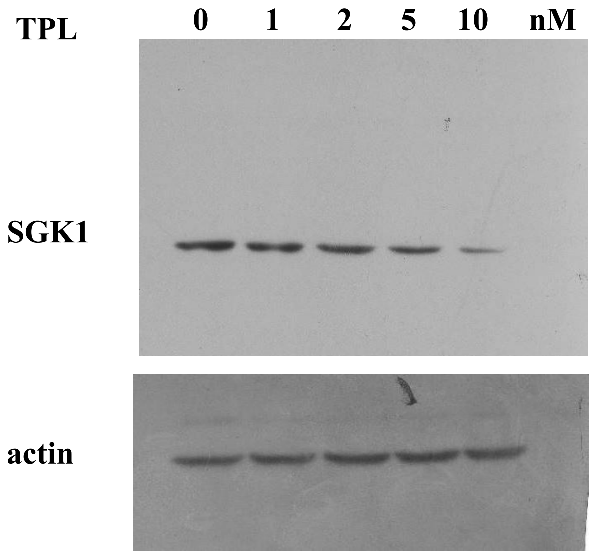

Figure S5 Original blots for Figure 7A

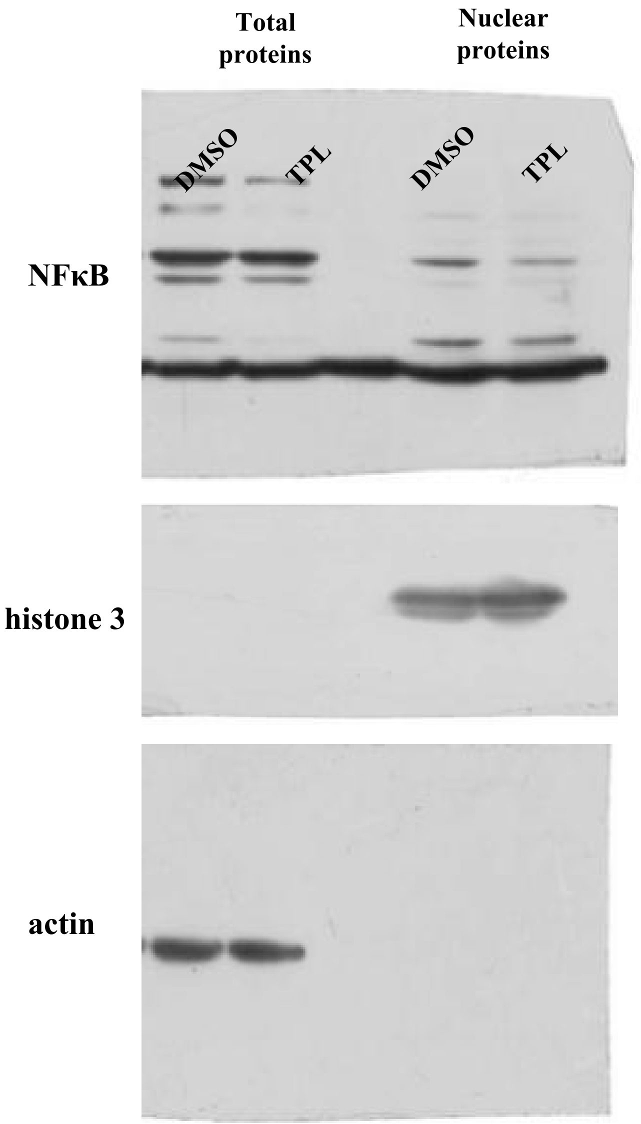

Figure S6 Original blots for Figure 8B and 8C

Figure 8B

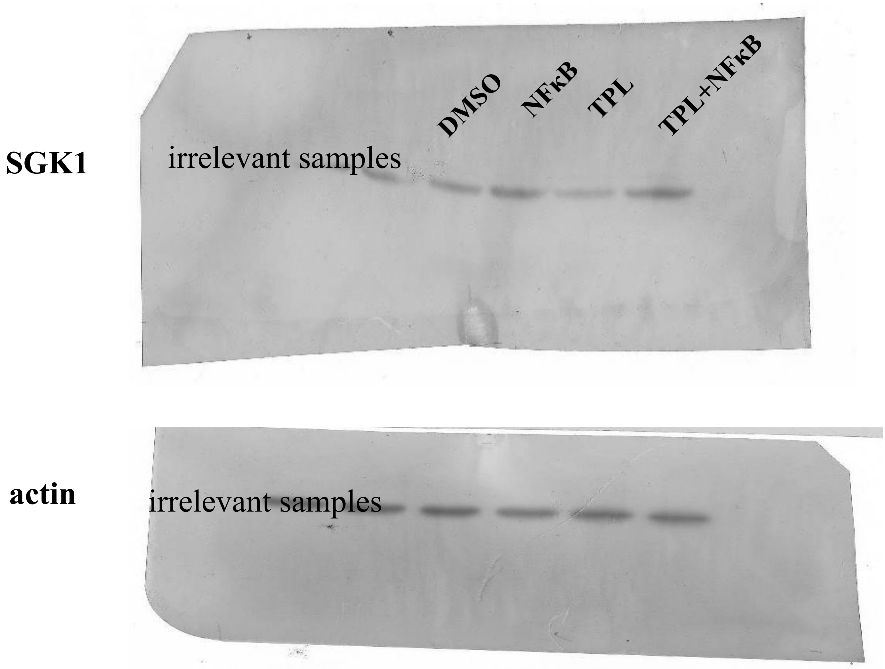

Figure 8C

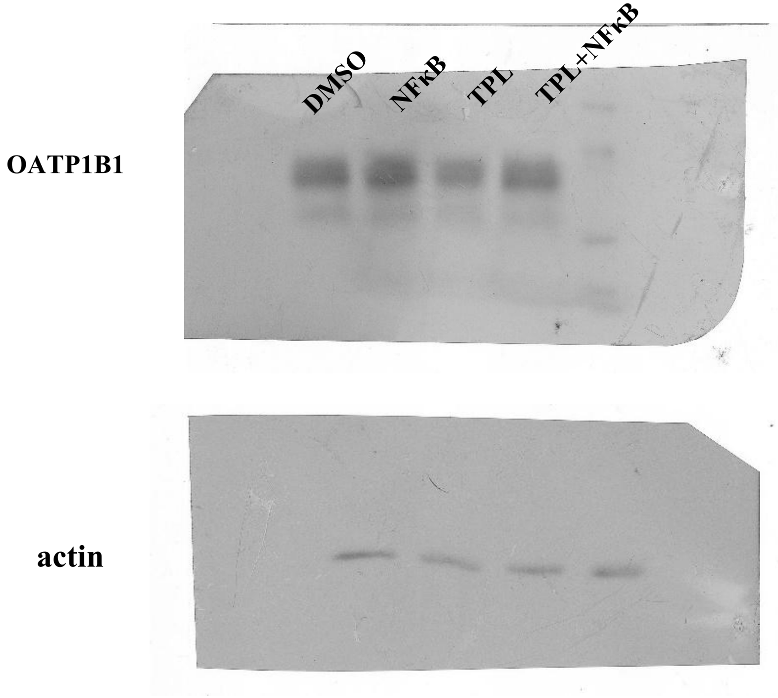

Supplement: Supplementary file 1 [file biology-14-01618-s001.zip › biology-3953602-supplementary.pdf]
